# Mechanism of action of curculigoside ameliorating osteoporosis: an analysis based on network pharmacology and experimental validation

**DOI:** 10.3389/fendo.2025.1549471

**Published:** 2025-08-21

**Authors:** Chuanfu Wei, Wenhuan Zhang, Chunbiao Lou, Nianhu Li, Hui Cao

**Affiliations:** ^1^ The First Clinical Medical College, Shandong University of Traditional Chinese Medicine, Jinan, China; ^2^ Affiliated Hospital of Shandong University of Chinese Medicine, Jinan, China

**Keywords:** curculigoside, osteoporosis, network pharmacology, molecular docking, micro CT technology

## Abstract

**Objective:**

This study aimed to predict and verify the mechanism of curculigoside in treating osteoporosis using network pharmacology, molecular docking technology, and micro-CT technology.

**Methods:**

Herb databases were searched to identify and screen potential targets of curculigoside. The GeneCards platform was utilized to mine osteoporosis-related targets. Cytoscape 3.6.0 software was employed to construct a compound-target-disease network. A protein–protein interaction (PPI) network for curculigoside in osteoporosis treatment was established, and core targets were screened. The Kyoto Encyclopedia of Genes and Genomes (KEGG) pathway enrichment and GO biological process analyses were performed using the Metascape database. Finally, molecular docking and micro-CT were used to validate core targets relevant to osteoporosis.

**Results:**

A total of 166 potential curculigoside targets and 4,313 osteoporosis-related targets were identified, with 91 common targets. Ten key targets, including matrix metalloproteinase (MMP)3, MMP9, interleukin (IL)-6, and caspase-3, were screened. KEGG pathway enrichment analysis indicated involvement in 10 pathways, such as the Rap1 signaling pathway and tumor necrosis factor (TNF) signaling pathway. Molecular docking results demonstrated strong binding affinity between curculigoside and the core targets. Micro-CT analysis revealed that curculigoside not only improved BMD, BV/TV, BS/BV, and Tb.Th but also reduced Tb.Sp in osteoporotic bone.

**Conclusions:**

Curculigoside is likely to treat osteoporosis through targets such as MMP3, MMP9, IL-6, and caspase-3, acting on signaling pathways including Rap1 and TNF. These results indicate that curculigoside exhibits multitarget and multipathway characteristics in osteoporosis treatment, providing a theoretical basis for further clinical investigation.

## 1 Introduction

Bone is a special type of connective tissue that is dynamically mineralized and has a variety of physiological functions (1, 2). Recognized for its mechanical properties, it serves as an attachment point for muscles, providing a structural basis and aiding movement (3, 4). Bone undergoes lifelong remodeling, including bone formation by osteoblasts and bone resorption by osteoclasts, which are essential for maintaining healthy bone mass (5–7). The entire process of bone remodeling is strictly controlled and coordinated by a variety of cells, including osteoblasts, which mediate bone formation, and osteoclasts, which mediate bone resorption (8, 9). Osteoblasts originate from mesenchymal stem cells in the bone marrow stroma and are responsible for the synthesis of the bone matrix and its subsequent mineralization (10, 11). Osteoclasts are large multinucleated giant cells formed by the fusion of mononuclear progenitors of monocytes/macrophages, and they are responsible for bone resorption (12, 13). The formation, proliferation, differentiation, and activity of these cells are controlled by local and systemic factors (14, 15).

Osteoporosis results from an imbalance between bone formation and resorption. It represents a common group of skeletal disorders characterized by destruction of bone microstructure, reduced bone mass, and increased bone fragility (16–18). The dynamic equilibrium between new bone formation by osteoblasts and old bone resorption by osteoclasts is crucial for maintaining bone tissue metabolism (19, 20). Osteoblasts, as the principal bone-forming cells, play a major role in the metabolic balance, growth, development, and repair of bone tissue (21, 22). During bone formation, osteoblasts undergo three stages: proliferation, differentiation, and apoptosis. These three stages involve: (1) the proliferation stage, where osteoblast precursor cells multiply; (2) the differentiation stage, where cells mature to secrete bone matrix; and (3) the apoptosis stage, where excess cells undergo programmed death to maintain tissue balance (23). Abnormalities in osteoblast proliferation, differentiation, or apoptosis play a critical role in the development of osteoporosis (24, 25).

Curculigoside, a naturally occurring phenolic compound, has been traditionally employed in many Asian countries for the treatment of osteoporosis (26). Its documented effects include antioxidant, anti-aging, immunomodulatory, and anti-inflammatory activities, along with the prevention of bone loss. Curculigoside demonstrates osteoprotective properties (27). Studies indicate that it improves bone microstructure and biomechanical properties while enhancing antioxidant enzyme activity in serum and bone tissue through the regulation of bone metabolic homeostasis (28, 29). Furthermore, curculigoside reduces bone loss, promotes osteogenesis, and inhibits adipogenesis in ovariectomized rats by upregulating endoplasmic reticulum-dependent bone morphogenetic protein-2 (BMP) (30). During aging, declining estrogen levels and excessive accumulation of reactive oxygen species (ROS) in bone tissue activate the nuclear factor kappa B (NF-κB) and MAPK pathways, inducing apoptosis and osteoclastogenesis (31). These findings suggest that curculigoside exerts bone-protective effects, though its precise mechanism of action remains unclear.

Traditional Chinese medicine is characterized by multiple chemical components, multiple targets, and multiple effects. Network pharmacology explores the complex relationships among drugs, targets, diseases, and pathways, enabling the identification of multiple components, targets, and signaling pathways. In this way, network pharmacology helps elucidate the therapeutic mechanisms of traditional Chinese medicine (32, 33). In this study, we applied a network pharmacological approach to identify potential targets of curculigoside and to elucidate its mechanism of action in the treatment of osteoporosis. Effective targets for curculigoside and osteoporosis were mined from multiple databases. Pathway enrichment and protein–protein interaction (PPI) network analyses were then performed on the overlapping curculigoside and osteoporosis to identify potential therapeutic pathways for osteoporosis treatment. To verify the protective effect of curculigoside on osteoporosis, *in vitro* validation was conducted by detecting the expression of cross-targets (e.g., IL-6, tumor necrosis factor alpha [TNF-α], matrix metalloproteinase (MMP)3, MMP9, caspase-3) in rat bone tissue using reverse transcription quantitative polymerase chain reaction (RT-qPCR) and enzyme-linked immunosorbent assay (ELISA). The results were consistent with the network pharmacology predictions.

## 2 Materials and methods

### 2.1 Animals

Thirty female SD rats of specific pathogen-free (SPF) grade, 8 weeks old, were purchased from Shandong Jinan Panyue Experimental Animal Co. Ltd. (Animal Production License No. SCXK [Lu] 20190003). All animals were housed under controlled, identical SPF standard environmental conditions (23°C ± 2°C, 12-h light/dark cycle), had free access to food, and were allowed free movement. The study protocol was approved by the Experimental Animal Ethics Committee of the Affiliated Hospital of Shandong University of Traditional Chinese Medicine (approval number: SDSZYYAWE20241105005).

### 2.2 Screening potential gene targets of curculigoside

Target gene data for curculigoside were collected from the Encyclopaedia of Traditional Chinese Medicine, SwissTargetPrediction (http://swisstargetprediction.ch), and the Similarity Ensemble Approach (https://sea.bkslab.org). The obtained gene targets were summarized and de-duplicated by searching the database using “cynarin” as the keyword. Target names were normalized using the UniProt database (https://www.uniprot.org/) (34).

### 2.3 Osteoporosis disease-target collection

The DisGeNET (https://www.disgenet.org/home/) and GeneCards (https://www.genecards.org/) (35) databases were used to search for relevant targets using the keyword “osteoporosis”. A network target map was constructed to identify overlapping targets between osteoporosis and the active ingredient. The identified overlapping targets are considered to play key roles in the antiosteoporosis activity of curculigoside.

### 2.4 Network construction

To elucidate the relationship among curculigoside, gene targets, and osteoporosis, Cytoscape 3.7.2 software was used to construct the curculigoside-target and osteoporosis-target networks (36). The drug-target network and disease-target network were established. In the network, nodes of different colors represent various drug and disease targets. Edges represent the relationships between two nodes, and their number, defined as the “degree,” determines the size of each node. A Venn diagram (http://bioinformatics.psb.ugent.be/webtools/Venn/) was then generated to identify the intersection of targets between curculigoside and osteoporosis, thereby revealing the potential targets of curculigoside against osteoporosis.

### 2.5 KEGG pathway enrichment and Gene Ontology analyses

The intersecting targets were used as inputs for the functional annotation tool DAVID 6.8 (https://david.ncifcrf.gov/). The identifier was set to OFFICIAL_GENE SYMBOL, and *Homo sapiens* and Gene List were selected. The Kyoto Encyclopedia of Genes and Genomes (KEGG) pathway enrichment and Gene Ontology (GO) enrichment analyses were then performed. *p* < 0.01 and FDR < 0.05 were set as the screening criteria to identify the biological processes and signaling pathways associated with Traditional Chinese Medicine (TCM) disease interventions.

### 2.6 Protein–protein interaction network construction

The potential targets of curculigoside in osteoporosis treatment were imported into the STRING 11.5 platform (https://cn.string-db.org/) (37). The species was set to *Homo sapiens* before conducting the PPI analysis. The PPI analysis output was saved in TSV file format, with the minimum interaction threshold set to 0.9. Cytoscape 3.7.2 was used to construct the PPI network and evaluate the node and edge degrees.

### 2.7 Molecular docking

Molecular docking was used to clarify the relationships among the potential curculigoside targets versus osteoporosis and the corresponding active ingredients. The 3D structure of the core target protein was retrieved from the RSCB PDB database (https://www.rcsb.org/) (38). The chemical structure of curculigoside was acquired from the PubChem database (https://pubchem.ncbi.nlm.nih.gov/) (39). AutoDock (https://vina.scripps.edu) was used to perform the molecular docking, and PYMOL (https://pymol.org/2/) was used to visualize the results.

### 2.8 Establishment of animal model

After 2 weeks of acclimatization, all female SD rats were randomly divided into three groups: sham-operated (sham) group, model (OVX) group, and curculigoside (curculigoside) group, with 10 rats in each group. Both the model group and the curculigoside group underwent bilateral ovariectomy via the abdominal approach, while the sham-operated group underwent excision of bilateral parietal ovarian adipose tissue. Penicillin was administered intraperitoneally to the rats daily for 3 days to prevent incisional infection. Penicillin at a concentration of 40,000 IU/mL was injected into rats at a volume of 1 mL/kg for 3 consecutive days to prevent infection (40). The rats in the curculigoside group were injected intraperitoneally at 7.5 mg/kg. In this study, three doses of Curculigoside were set: 5, 7.5, and 10 mg/kg. Based on hematoxylin–eosin (HE) staining and TRAP staining to observe the expression of bone trabeculae and osteoclasts, no significant difference was found between the 7.5- and 10-mg/kg intervention groups. Therefore, 7.5 mg/kg was selected as the optimal dose. The experimental data are provided in **Supplementary File 1**. All rats were treated continuously for 12 weeks, and bone mineral density testing was performed.

### 2.9 HE staining and SOFG staining

After fixation, decalcification, and paraffin embedding, HE staining was performed, and the pathological structure of the bone tissue was observed following dehydration and clearing. Safranine O and Fast Green (SOFG) staining was carried out after dewaxing and hydration of the sections. The sections were sequentially stained with hematoxylin, solid green, and mesene O, followed by dehydration and clearing, to more precisely display structural changes such as trabeculae and bone marrow cavities. For the liver and kidney tissues of rats, the HE staining procedure was as follows: the tissues were fixed and embedded in paraffin, then sectioned. After dewaxing and hydration, the sections were stained with hematoxylin–eosin. Following dehydration and clearing, the tissue structures were observed to evaluate whether any of the treatment groups caused toxic damage to the liver and kidneys.

### 2.10 Microcomputed tomography

The removed femur was placed in 4% paraformaldehyde (4°C, 24 h) for fixation. The microstructure of the femur was scanned and analyzed using the SkyScan imaging system. Each sample was carefully placed so that the femoral stem was oriented as vertically as possible. Bone morphometric parameters of the femur were obtained, including bone mineral density (BMD) on total volume, bone surface area to bone volume ratio (BS/BV), bone surface area to tissue volume ratio (BS/TV), trabecular thickness (Tb.Th), and trabecular separation (Tb.Sp).

### 2.11 Measurement of inflammatory cytokines

The plasma of mice was centrifuged, and the supernatant was collected to determine the levels of inflammatory factors. The expression levels of IL-6, TNF-α, IL-1β, osteocalcin (OCN), and insulin-like growth factor-1 (IGF-1) were measured using ELISA kits according to the manufacturer’s instructions.

### 2.12 RNA isolation and real-time PCR

Total RNA was extracted from rat bone tissue using the Spark Jade Science & Technology Co., Ltd. (SPARK) easy Improved Tissue/Cell RNA Kit (Spark Jade, AC0202). RNA reverse transcription was performed using the SPARK script IIRT Plus Kit (Spark Jade, AG0304) according to the manufacturer’s instructions. qPCR reactions, prepared with 2 × SYBR Green qPCR Mix (Spark Jade, AH0104), were run at 94°C for 3 min. Primers for collagen I, IL-6, TNF-α, MMP3, MMP9, and caspase-3 were used for the RT-qPCR. Glyceraldehyde-3-phosphate dehydrogenase (GAPDH) served as the internal reference. The relative expression levels of the target and reference genes were quantified using the 2^−ΔΔCT^ method (**Table 1**).

**Table 1:** Primers in qRT-PCR.

Name	Primer	Sequence
Collagen I	Forward	ACTGGTACATCAGCCCGAAC
	Reverse	AATCCATCGGTCATGCTCTC
Caspase-3	Forward	AGCATGAAAGGGTGGTCTCA
	Reverse	GICGGCATACTGTTTCAGCA
MMP3	Forward	GGGTCTCTTTCACTCAGCCAACAC
	Reverse	ACAGGCGGAACCGAGTCAGG
MMP9	Forward	CGTCTTCCAGTACCGAGAGAAAGC
	Reverse	TIGGTCCACCTGGTTCAACTCAC
TNF-α	Forward	CCCCAAAGGGATGAGAAGTT
	Reverse	GGTCTGGGCCATAGAACTGA
IL-6	Forward	ATG AAC TCC TTC TCC ACA AG
	Reverse	GTG CCT GCA GCT TCG TCA GCA
GAPDH	Forward	TCACGACCATGGAGAAGGCT
	Reverse	CAGGAGGCATTGCTGATGATC

### 2.13 Statistical analysis

**Figure 1:**
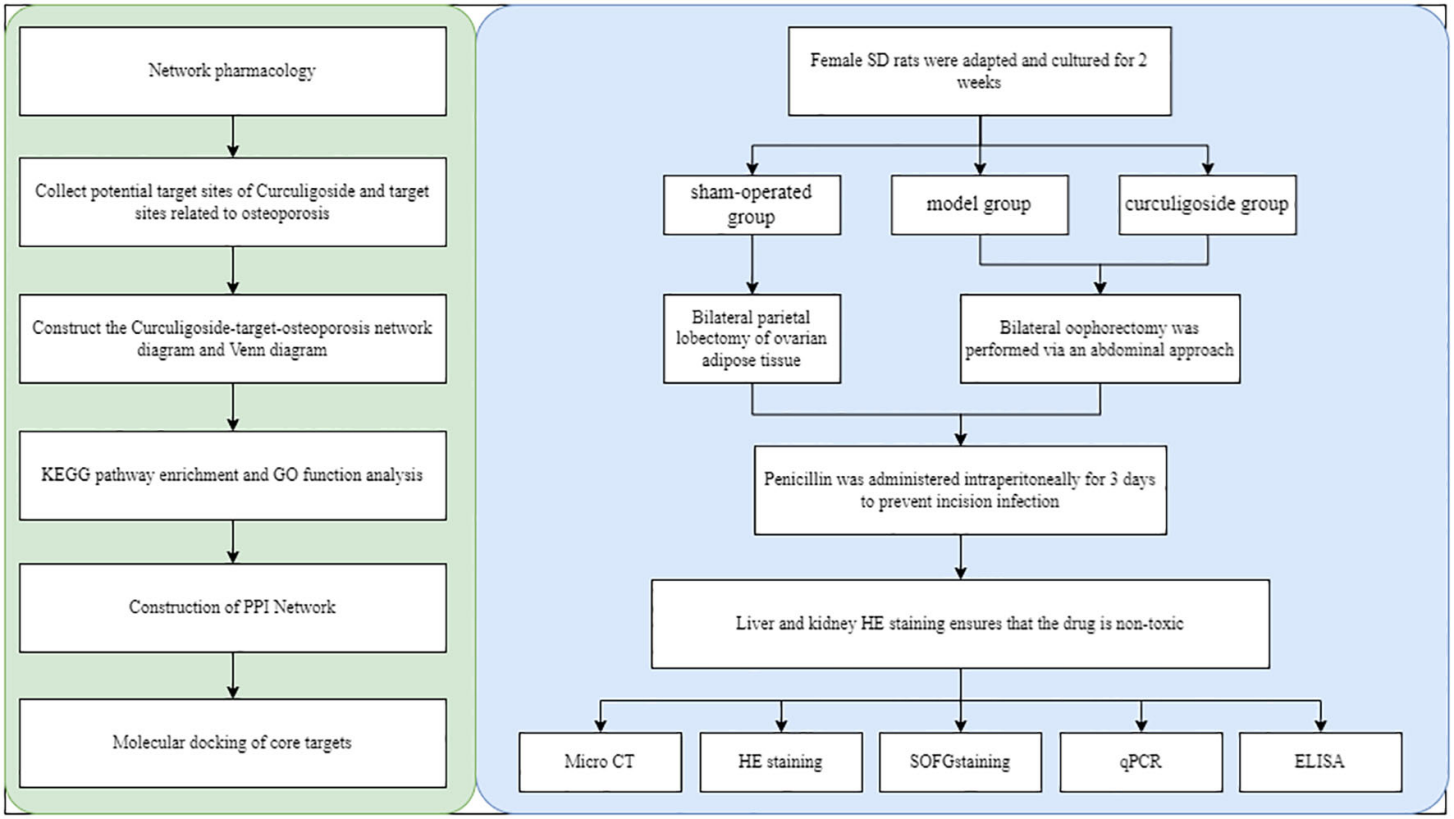
Flowchart of the research.

All data were analyzed with GraphPad Prism v.8 (GraphPad Software, La Jolla, CA, USA). One-way analysis of variance (ANOVA) was used to detect significant differences between groups. *p* < 0.05 indicated statistical significance. **Figure 1** shows the overall flowchart of the research.

## 3 Results

### 3.1 Potential targets of curculigoside against osteoporosis

**Figure 2:**
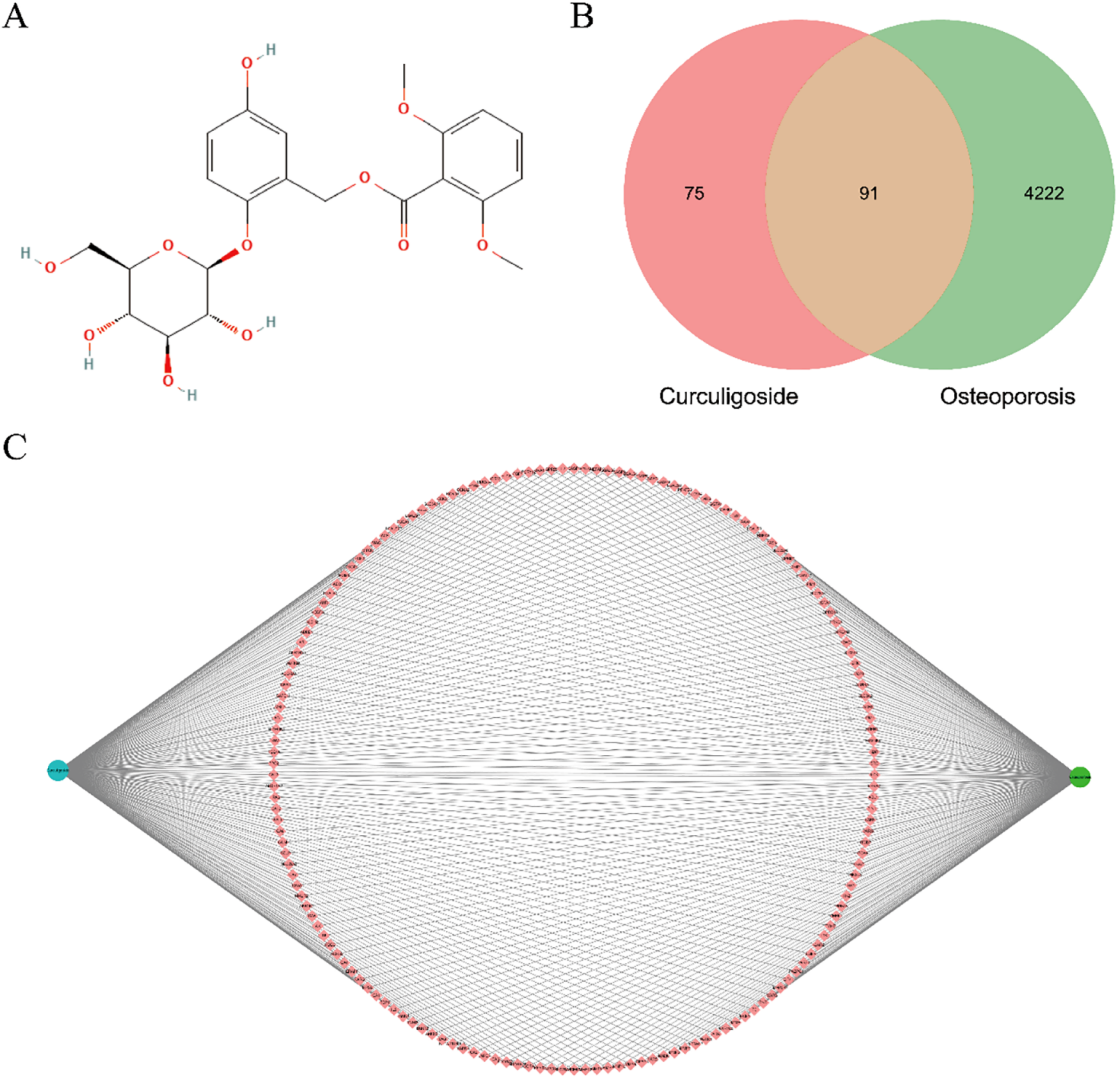
Network target diagram and targets common to curculigoside and osteoporosis. **(A)** Molecular structure of curculigoside. **(B)** Network target diagram. **(C)** Venn diagram of shared targets.

The chemical molecular formula of curculigoside is C_22_H_26_O_11_, and its molecular structure is shown in **Figure 2A**. A total of 188 potential targets were obtained by searching the Encyclopaedia of Chinese Medicine database, SwissTargetPrediction, and the Similarity Ensemble Approach database. A total of 4,313 osteoporosis-related targets were retrieved from the DisGeNET and GeneCards databases. By linking the two groups of targets, 91 overlapping targets were identified as valid targets for the treatment of osteoporosis with curculigoside. Finally, a network target map was constructed using Cytoscape (3.7.1) software and Venny (2.1.0) for the 91 valid targets (**Figures 2B, C**).

### 3.2 Construction of a protein–protein interaction network

**Figure 3:**
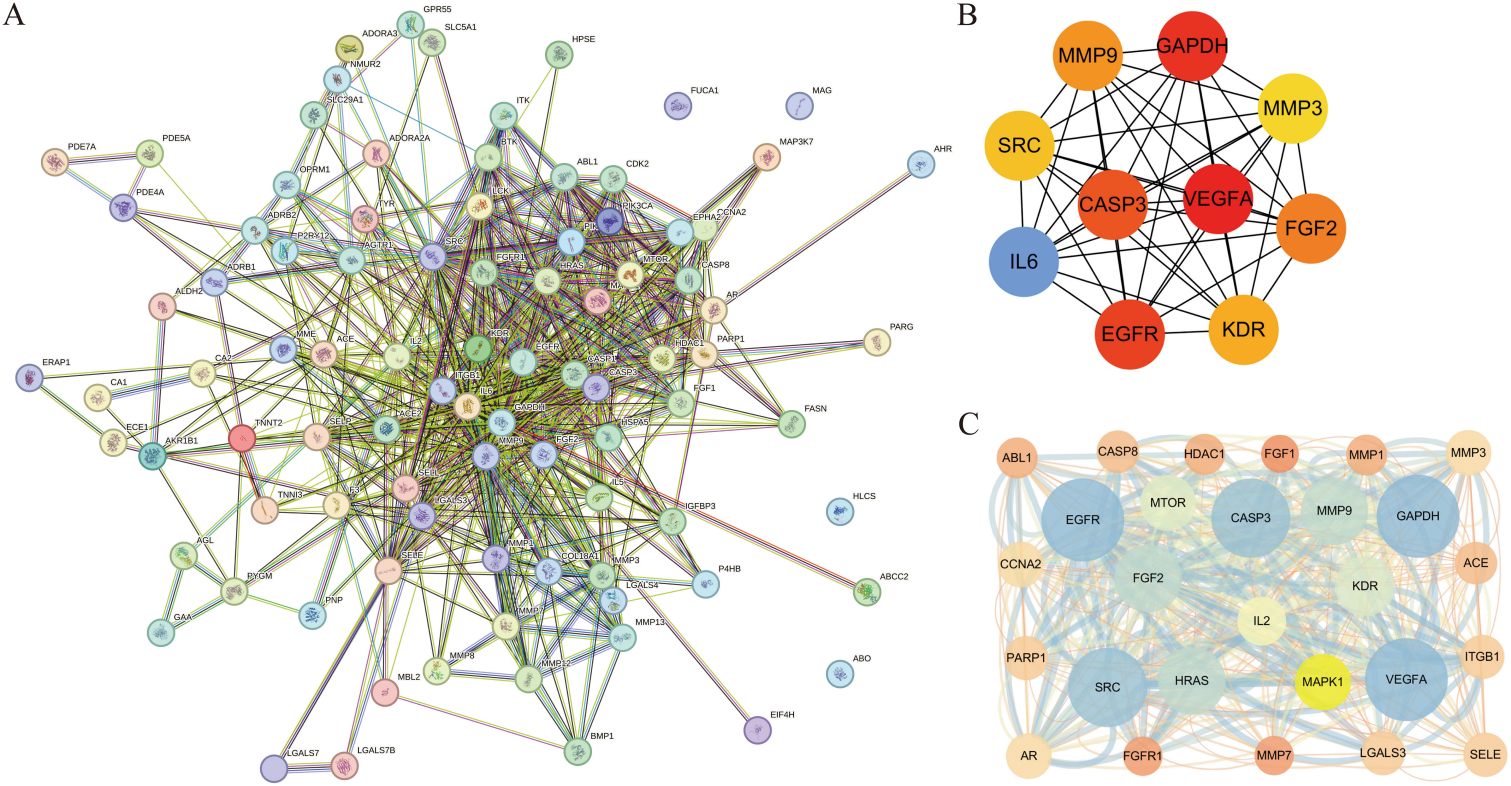
Construction of protein–protein interaction (PPI) network and disease topology analysis. **(A)** PPI network of overlapping targets. **(B)** Intersecting genes from the STRING database were constructed using Cytoscape. **(C)** Clustering analysis.

The validated targets of cenobacterial glycosides for osteoporosis were imported into the STRING (11.5) database, set to “*Homo sapiens*”, and subjected to PPI analysis. The PPI data were imported into Cytoscape software, and topological and clustering analyses were performed using the Cytoscape plug-in cytoHubba and MCODE. The top 10 central genes obtained from the topological analysis were Vascular Endothelial Growth Factor A (VEGFA), GAPDH, Epidermal Growth Factor Receptor (EGFR), CASP3, IL6, Fibroblast Growth Factor 2 (FGF2), MMP9, Kinase Insert Domain Receptor (KDR), SRC, and MMP3. Among these genes, Vascular Endothelial Growth Factor A (VEGFA) acts as an active growth factor in angiogenesis and endothelial cell growth; it induces endothelial cell proliferation, promotes cell migration, and inhibits apoptosis. GAPDH possesses 3-phosphoglyceraldehyde dehydrogenase and nitrosylase activities, which play roles in glycolysis and nuclear function, respectively. FGF2 plays an important role in the regulation of cell survival, cell division, cell differentiation, and cell migration. KDR and SRC play an important role in the regulation of angiogenesis and vascular permeability. MMP9 and MMP3 play an important role in local protein hydrolysis and leukocyte migration in the extracellular matrix, as well as in osteoclast-mediated bone resorption (**Figures 3A, B**). The results of the clustering analysis are shown in **Figure 3C**.

### 3.3 KEGG enrichment and GO functional analyses

**Figure 4:**
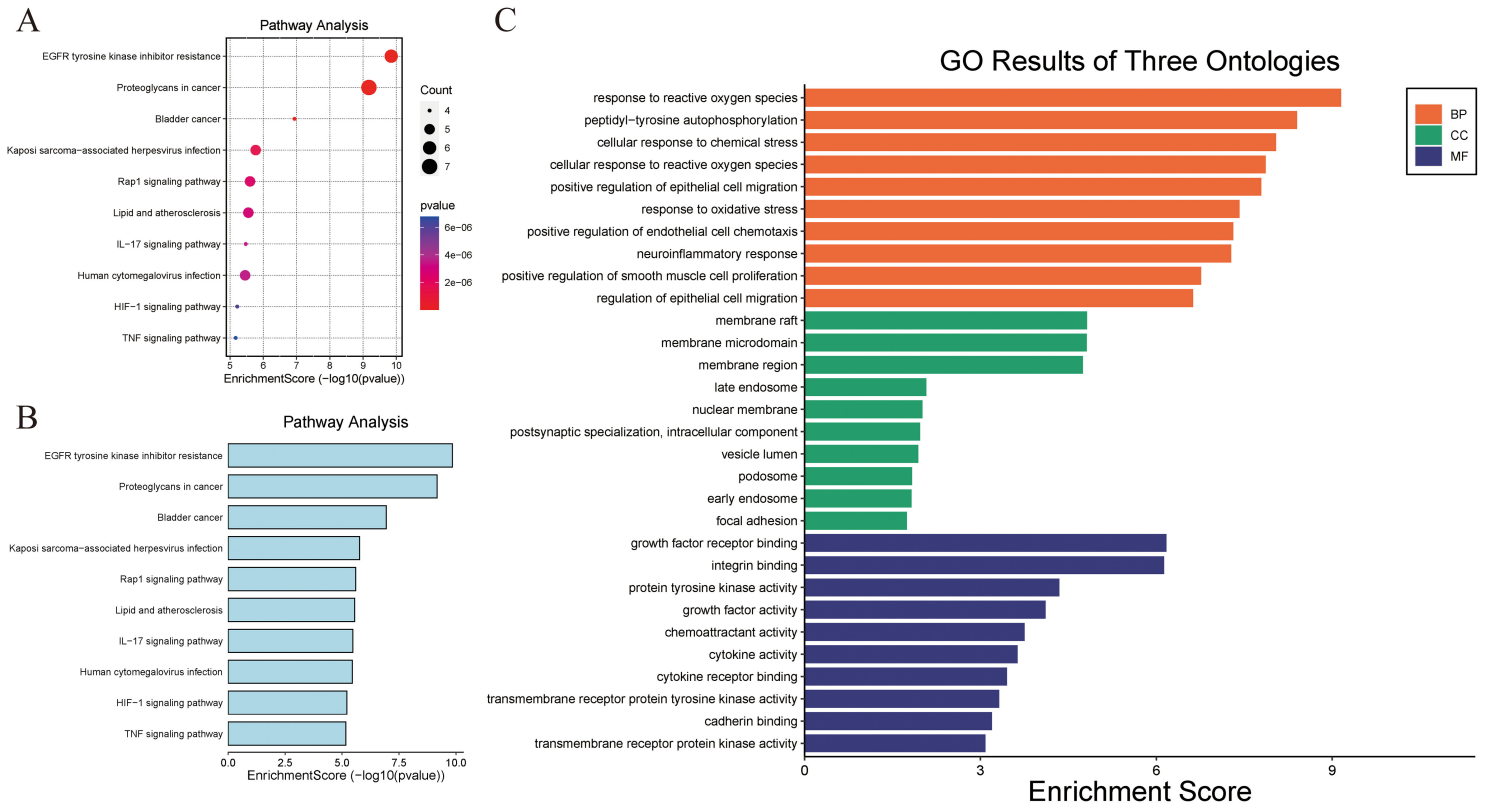
KEGG and GO functional enrichment analyses. **(A, B)** KEGG pathway enrichment analysis bubble map. **(B)** GO Ontology analysis.

A total of 69 signaling pathways were identified by GO and KEGG functional enrichment analyses of the validated targets using Metascape software. Using a threshold of *p* < 0.01 and sorting by ascending *p*-value, 47 KEGG pathways were obtained, with the top 10 shown in **Figure 4**. Further analysis of GO functional enrichment results, using the same screening criteria (*p* < 0.01 and ascending *p*-value), yielded a total of 514 BPs, 5 CCs, and 31 MFs, as shown in **Figure 4**. **Supplementary File 2** provides the detailed GO analysis results, while **Supplementary File 3** presents the specific KEGG analysis outcomes.

### 3.4 Molecular docking

**Figure 5:**
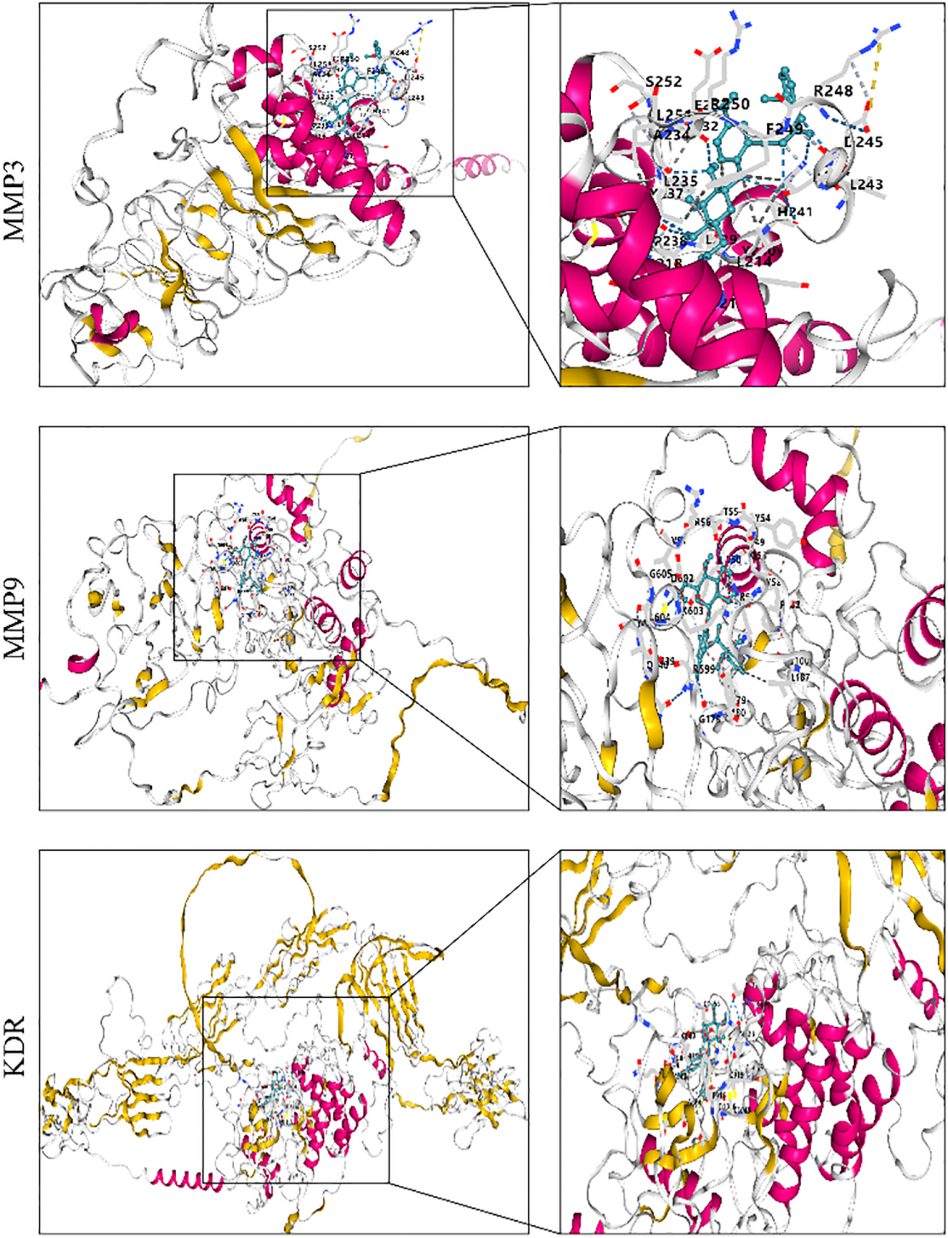
3D molecular docking of the active ingredient with target proteins.

Molecular docking analysis was used to assess the binding ability of curculigoside to key proteins. To evaluate its potential antiosteoporotic effect, molecular docking was performed between curculigoside and the key proteins VEGFA, GAPDH, EGFR, CASP3, IL-6, FGF2, MMP9, KDR, SRC, and MMP3. A lower binding energy indicates higher docking affinity and stronger binding ability. The molecular docking results showed binding energies ranging from − 5.8 to − 8.2. Targets with binding energies ≤ − 6 were considered potentially active, while those ≤ − 8 indicated strong stability and activity. Among them, curculigoside exhibited strong binding affinity to MMP3, MMP9, and KDR with binding energies ≤ − 8, suggesting high binding stability and potential biological activity (41) (as shown in **Figure 5**).

### 3.5 Liver and kidney tests show no toxicity

**Figure 6:**
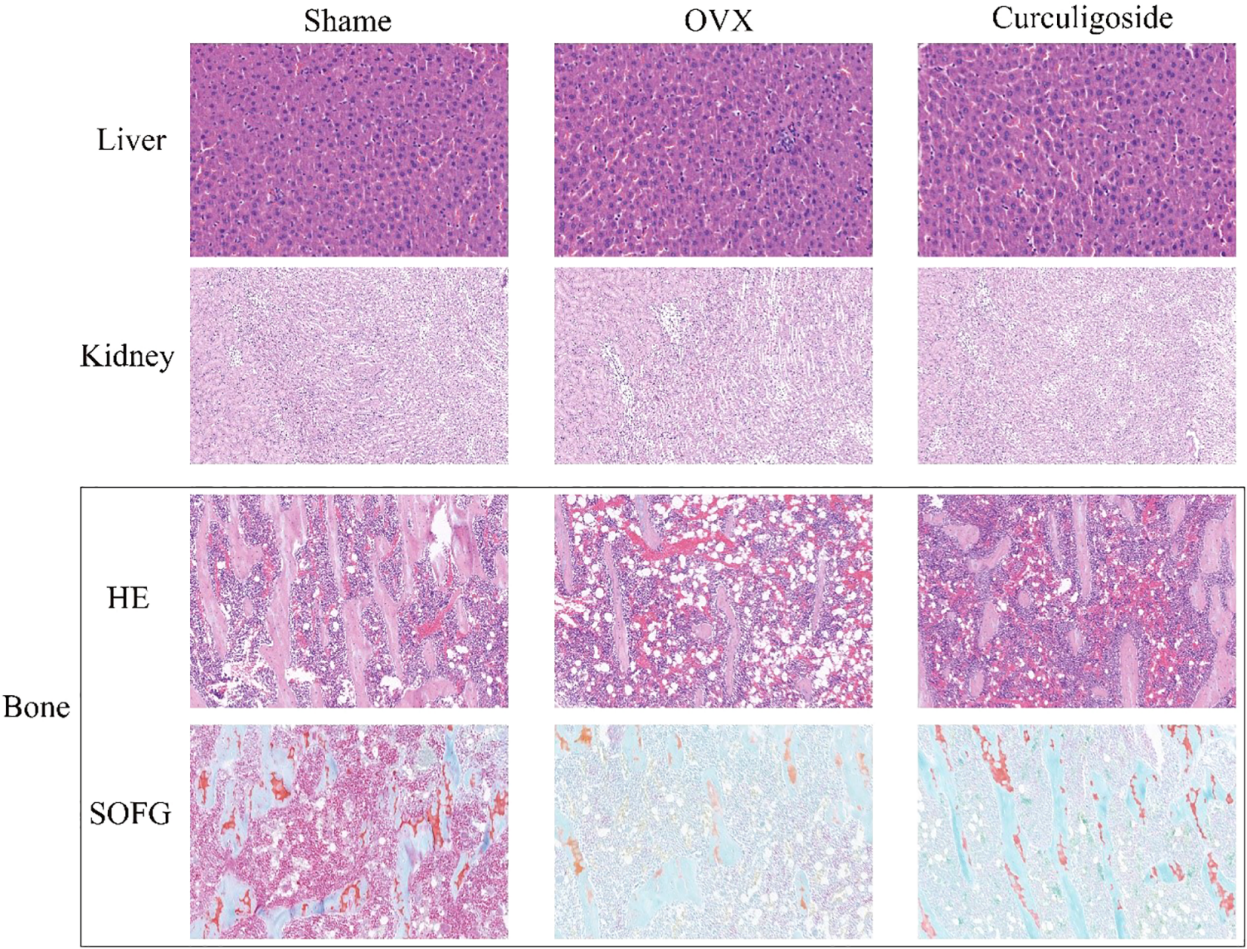
Histopathological sections of the liver, kidney, and bone tissues of rats in each group (H&E and SOFG staining).

**Figure 6** shows that the liver and kidney tissue structures in the sham group, OVX group, and curculigoside group were all normal. No lesions such as steatosis, necrosis, inflammatory cell infiltration, or renal tubular epithelial cell injury were observed, indicating that the treatments did not cause liver or kidney toxicity. In bone tissue, the results of HE staining and SOFG staining confirmed the successful establishment of the osteoporosis model in the OVX group, which exhibited pathological features such as sparse and fractured trabeculae and an enlarged bone marrow cavity. The trabecular bone structure in the curculigoside group was improved compared to that in the OVX group and was closer to the normal state, indicating that curculigoside has a therapeutic effect on osteoporosis.

### 3.6 Bone density and CT evaluation

**Figure 7:**
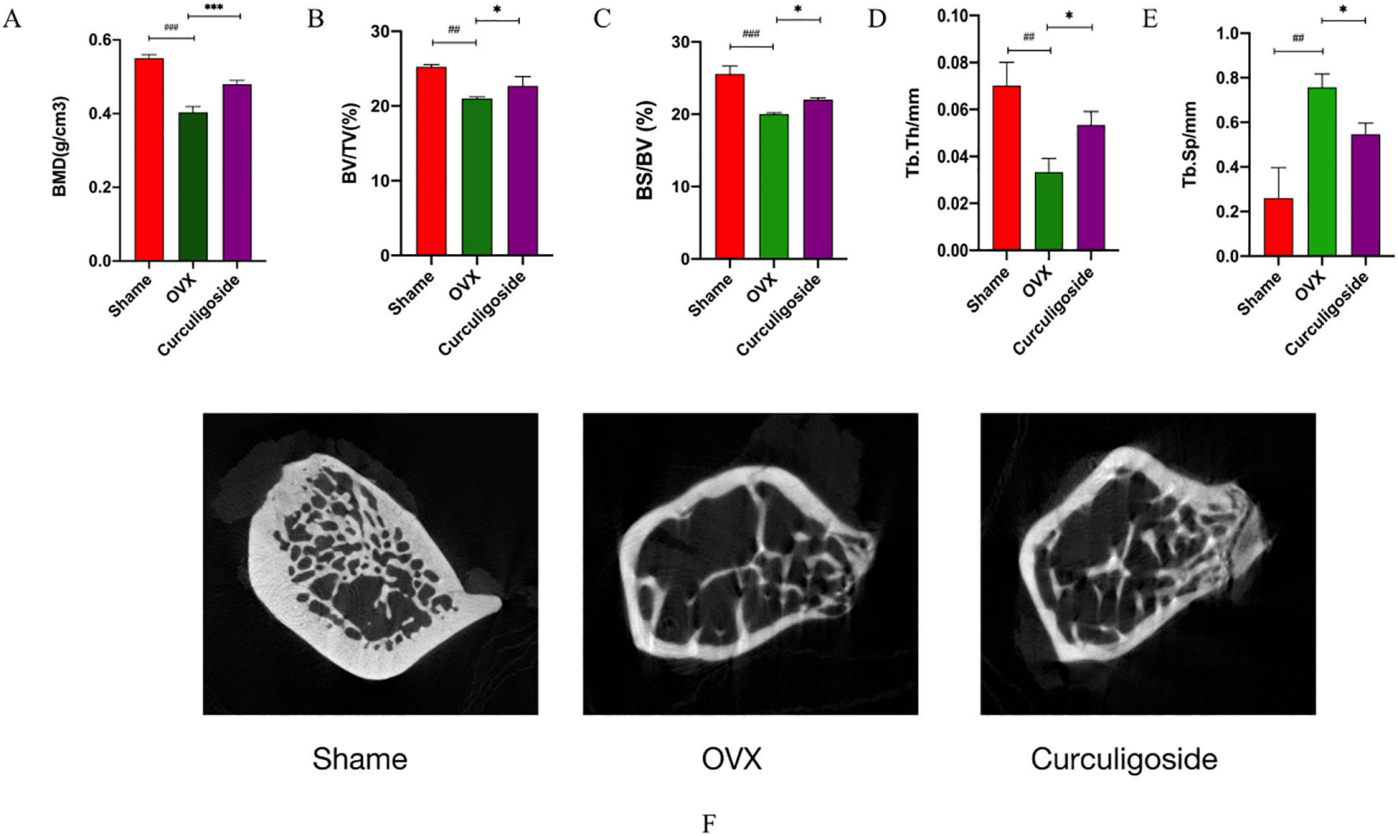
Detection of bone tissue by micro-CT. **(A–E)** Bone tissue-related index measurements. **(F)** CT images. *n* = 3, ^*^ *p* < 0.05; ^**^ *p* < 0.01. ^#^p < 0.05; ^##^p < 0.01; ^###^p < 0.01.

Results from the OVX and curculigoside groups showed a significant decrease in femoral bone mineral density compared to the sham group. The curculigoside group showed a significant increase in BMD compared to the OVX group (*p* < 0.05). Daily administration of cenicaloside further increased femoral bone density. 2D micro-CT scans revealed the trabecular bone microstructure at the femoral epiphysis after 12 weeks of curculigoside treatment. Quantitative parameters included BMD, BV/TV, BS/BV, Tb.Sp, and Tb.Th. Cenchoside treatment positively affected all CT parameters (**Figure 7**).

### 3.7 The effect of curculigoside on inflammatory and proapoptotic factors

**Figure 8:**
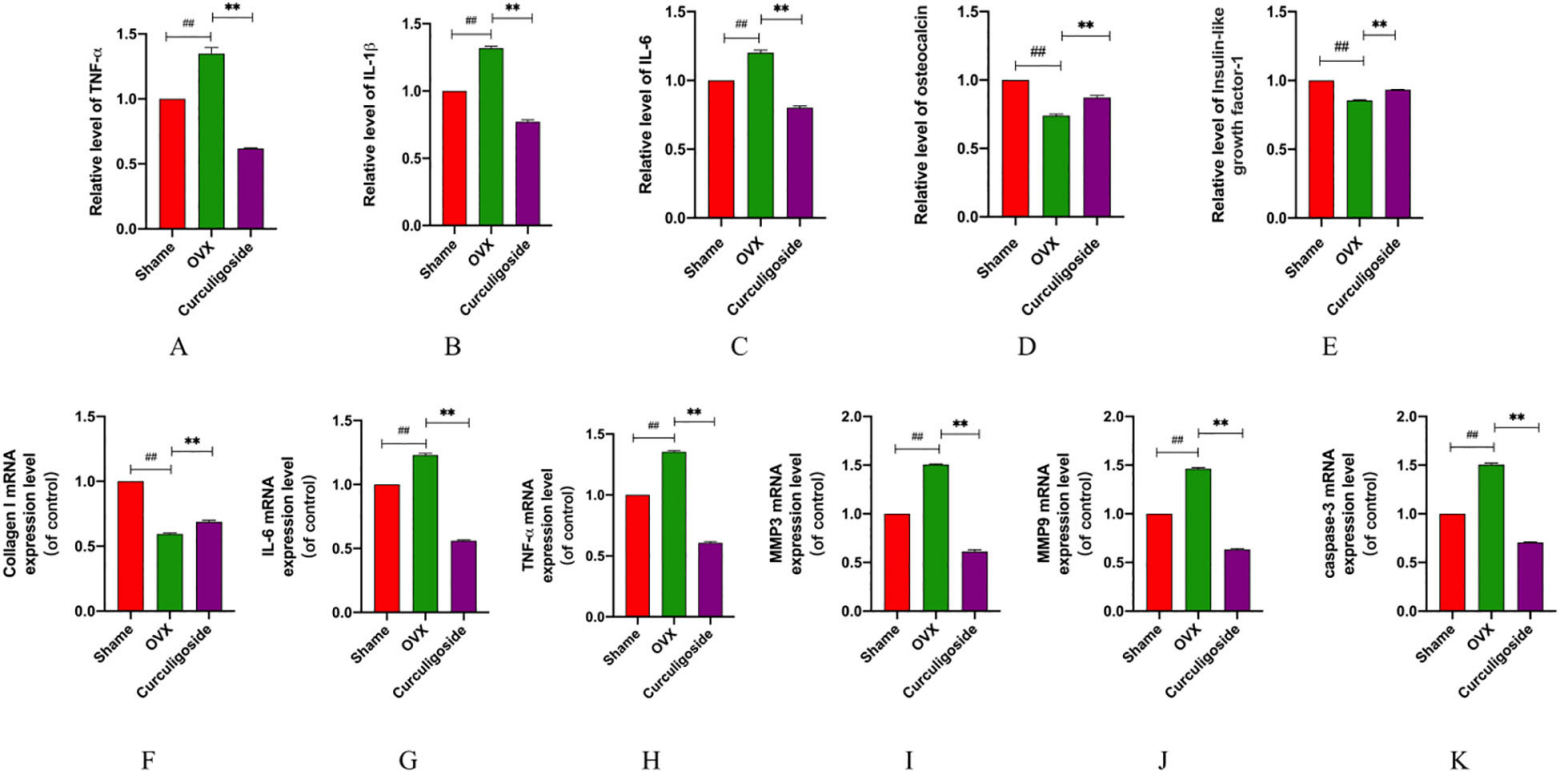
Detection of inflammatory and proapoptotic factors. **(A–E)** The expression levels of IL-6, IL-1β, TNF-α, OCN, and IGF-1 were measured by ELISA. **(F–K)** mRNA levels of IL-6, TNF-α, MMP3, MMP9, caspase-3, and collagen (I) n = 3, *p < 0.05; **p < 0.01; ***p < 0.01. ^#^p < 0.05; ^##^p < 0.01; ^###^p < 0.01.

Collagen I, a major constituent of the extracellular matrix, is sensitive to external stimulation. RT-qPCR was used to detect the messenger RNA (mRNA) expression of collagen I, IL-6, TNF-α, MMP3, MMP9, and caspase-3, while ELISA kits were employed to measure the levels of IL-6, TNF-α, IL-1β, OCN, and IGF-1. The results demonstrated that, in the OVX group, collagen I mRNA expression was significantly downregulated and OCN and IGF-1 levels were reduced. Conversely, the mRNA levels of IL-6, TNF-α, MMP3, MMP9, and caspase-3, as well as the secretion levels of IL-6, TNF-α, and IL-1β, were significantly increased. Treatment with curculigoside reversed these changes by upregulating collagen I mRNA and increasing OCN and IGF-1 levels, while downregulating the mRNA and protein levels of IL-6, TNF-α, MMP3, MMP9, and caspase-3 (**Figure 8**).

## 4 Discussion

Osteoporosis is a common skeletal metabolic disease characterized primarily by reduced bone mass. In recent years, its prevalence has risen significantly and has increasingly affected younger populations. Consequently, identifying improved treatments has become a major focus of current research. Cyberpharmacology enables comprehensive visualization and analysis of drug chemistry, disease targets, and pathways of action, facilitating the exploration of curculigoside’s potential mechanisms for treating osteoporosis (42, 43). Employing network pharmacology and molecular docking techniques, this study enabled the initial identification of curculigoside’s potential targets and complex molecular mechanisms in osteoporosis.

A Venn diagram of the “curculigoside-target-disease” network was constructed, identifying 166 cross-targets and 10 related signaling pathways. These pathways are primarily involved in the negative regulation of reactive oxygen species, oxidative stress, and cell proliferation and differentiation, through the regulation of the Rap1 signaling pathway, interleukin (IL)-17 signaling pathway, and Hypoxia-Inducible Factor 1 (HIF-1) signaling pathway. The IL-17, HIF-1, and TNF signaling pathways may contribute to curculigoside’s therapeutic effects in osteoporosis. The protein–protein interaction network was analyzed using Cytoscape 3.6.0, identifying 10 core targets. Curculigoside may exert its therapeutic effect on osteoporosis by binding to these key targets. The results showed that curculigoside reduced the mRNA expression of IL-6, TNF-α, and IL-1β; decreased the damage caused by inflammatory factors to osteoblasts; downregulated the expression of MMP3, MMP9, and caspase-3; and upregulated the expression of the collagen I gene. It also reduced the secretion levels of IL-6, IL-1β, and TNF-α; increased the secretion levels of OCN and IGF-1; improved the status of type I collagen; promoted the proliferation of osteoblasts; and slowed the progression of osteoporosis. In this study, the downregulation of IL-6, TNF-α, IL-1β, MMP3, MMP9, and caspase-3 by curculigoside aligns with previous findings that these factors mediate osteoblast injury and extracellular matrix degradation in osteoporosis (44–46). For example, Min et al. demonstrated that IL-17-driven inflammation promotes osteoclastogenesis and osteoblast apoptosis, which is consistent with our observation of reduced proinflammatory cytokine levels. Additionally, the upregulation of collagen I, OCN, and IGF-1 by curculigoside supports its role in enhancing osteoblast function, paralleling the reported mechanisms of VEGFA and FGF2 in promoting bone formation (46). These results collectively suggest that curculigoside may inhibit osteoclast differentiation and promote osteoblast proliferation through multitarget regulation of inflammatory and oxidative stress pathways. We suggest that cynarin may be involved in the regulation of ROS-related biological processes, estrogen signaling, osteoblast apoptosis, and osteoclast differentiation pathways in the treatment of osteoporosis. Both osteoclast and osteoblast differentiation, as well as their proliferation, are closely related to reactive oxygen species (47). ROS can cause severe damage to bone tissue by accelerating bone resorption, which is strongly associated with the upregulation of osteoclast differentiation via the NF-κB and calcium-regulated neurophosphatase pathways (48). Cynarin processes multiple biological activities, such as antioxidant and anti-inflammatory properties, which have been demonstrated in both animal and cellular experiments (26, 27). Liu et al. (49) suggested that cynarin could attenuate oxidative stress and inhibit osteoclastogenesis and MMP9-specific gene expression by modulating the Nrf2/NF-κB signaling pathway in RAW264.7 cells. This is consistent with our KEGG and GO enrichment results, which highlighted the involvement of NF-κB and ROS-related pathways in curculigoside’s mechanism, further validating its antiosteoporotic effects through the regulation of oxidative stress and inflammation. Mitochondrial impairment exacerbates the accumulation of reactive oxygen species, leading to oxidative stress and activation of osteoclast activity, as well as increased expression of MMPs and secretion of inflammatory factors through NF-κB activation. The results of KEGG and GO functional enrichment suggest that curculigoside may treat osteoporosis by inhibiting the overproduction of osteoclastic cytokines and their induction of oxidative damage, primarily through modulation of the Rap1, IL-17, HIF-1, and TNF signaling pathways. This modulation may reduce oxidative damage, restore mitochondrial dysfunction, decrease osteoblast apoptosis, and help maintain the dynamic balance between bone resorption and bone formation.

In this study, micro-CT imaging was employed to assess histopathological changes in the bone tissues of ovariectomized rats, providing both quantitative and qualitative evidence for curculigoside’s effects on bone microarchitecture. The technique helped confirm that curculigoside improved bone density and microstructural parameters (e.g., trabecular thickness and connectivity) (50–52), supporting the study’s overall conclusion on the drug’s antiosteoporotic efficacy. However, it should be noted that micro-CT alone cannot fully characterize cellular and molecular mechanisms. Future studies could combine histomorphological analysis and immunohistochemistry to further validate these findings.

## 5 Conclusion

This study combined network pharmacology, molecular docking, and *in vivo* experiments to investigate curculigoside’s mechanism against osteoporosis. Network analysis identified 91 shared targets (e.g., MMP3, MMP9, IL-6, caspase-3) enriched in the Rap1 and TNF signaling pathways. Molecular docking showed strong binding affinity (≤ − 8 kcal/mol) to core targets. In ovariectomized rats, curculigoside improved bone parameters (BMD, BV/TV, Tb.Th↑; Tb.Sp↓) and regulated inflammatory (IL-6, TNF-α↓) and osteogenic (collagen I, IGF-1↑) markers. These results indicate that curculigoside exerts multitarget, multipathway effects on bone remodeling, inflammation, and apoptosis, providing a basis for its clinical use.

## Data Availability

The original contributions presented in the study are included in the article/**Supplementary Material**. Further inquiries can be directed to the corresponding author.
