# Correction: Mechanism of action of curculigoside ameliorating osteoporosis: an analysis based on network pharmacology and experimental validation

**DOI:** 10.3389/fendo.2025.1690095

**Published:** 2025-09-09

**Authors:** Chuanfu Wei, Wenhuan Zhang, Chunbiao Lou, Nianhu Li, Hui Cao

**Affiliations:** ^1^ The First Clinical Medical College, Shandong University of Traditional Chinese Medicine, Jinan, China; ^2^ Affiliated Hospital of Shandong University of Traditional Chinese Medicine, Jinan, China

**Keywords:** curculigoside, osteoporosis, network pharmacology, molecular docking, micro CT technology

Affiliation “1 The First Clinical Medical College, Shandong University of Traditional Chinese Medicine, Jinan, China” and was erroneously given as “1 Shandong University of Chinese Medicine, Jinan, China”.

The original version of this article has been updated.

