# Supplementary figures and images for: Mechanism of action of curculigoside ameliorating osteoporosis: an analysis based on network pharmacology and experimental validation
Source: Front Endocrinol (Lausanne). 2025 Aug 21;16:1549471. doi: 10.3389/fendo.2025.1549471 (PMC12408278; doi:10.3389/fendo.2025.1549471)

# Shame

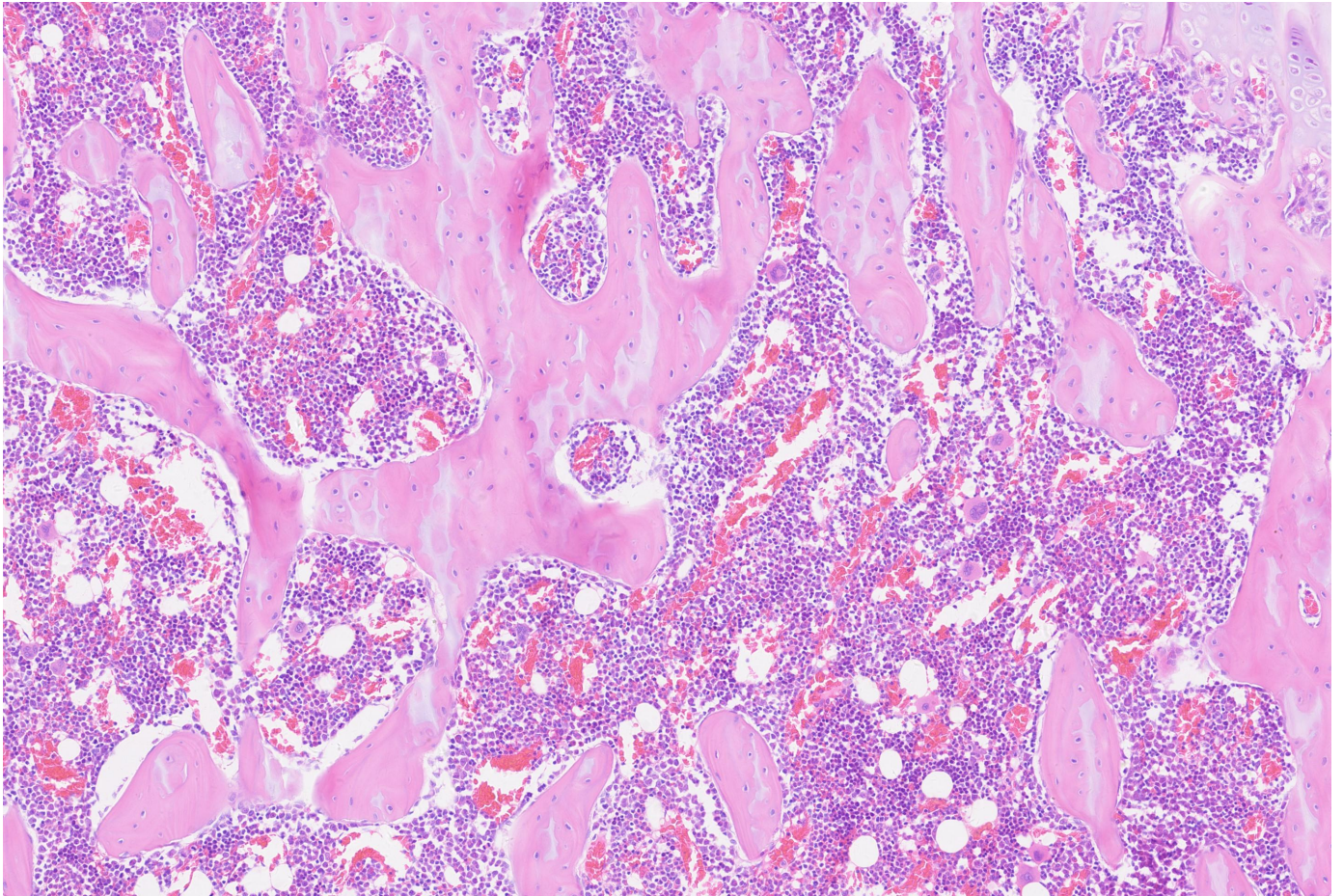

OVS

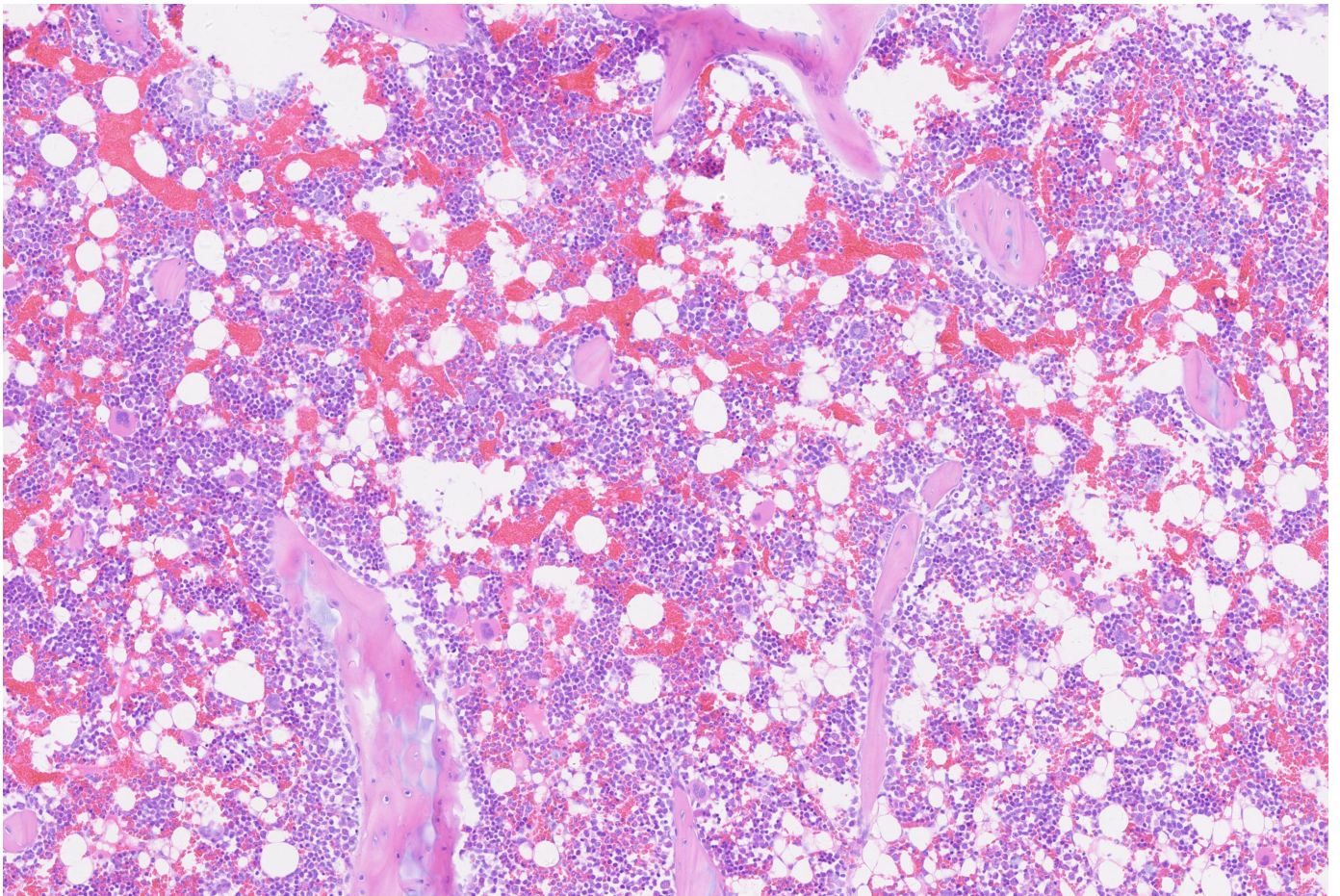

5mg/kg

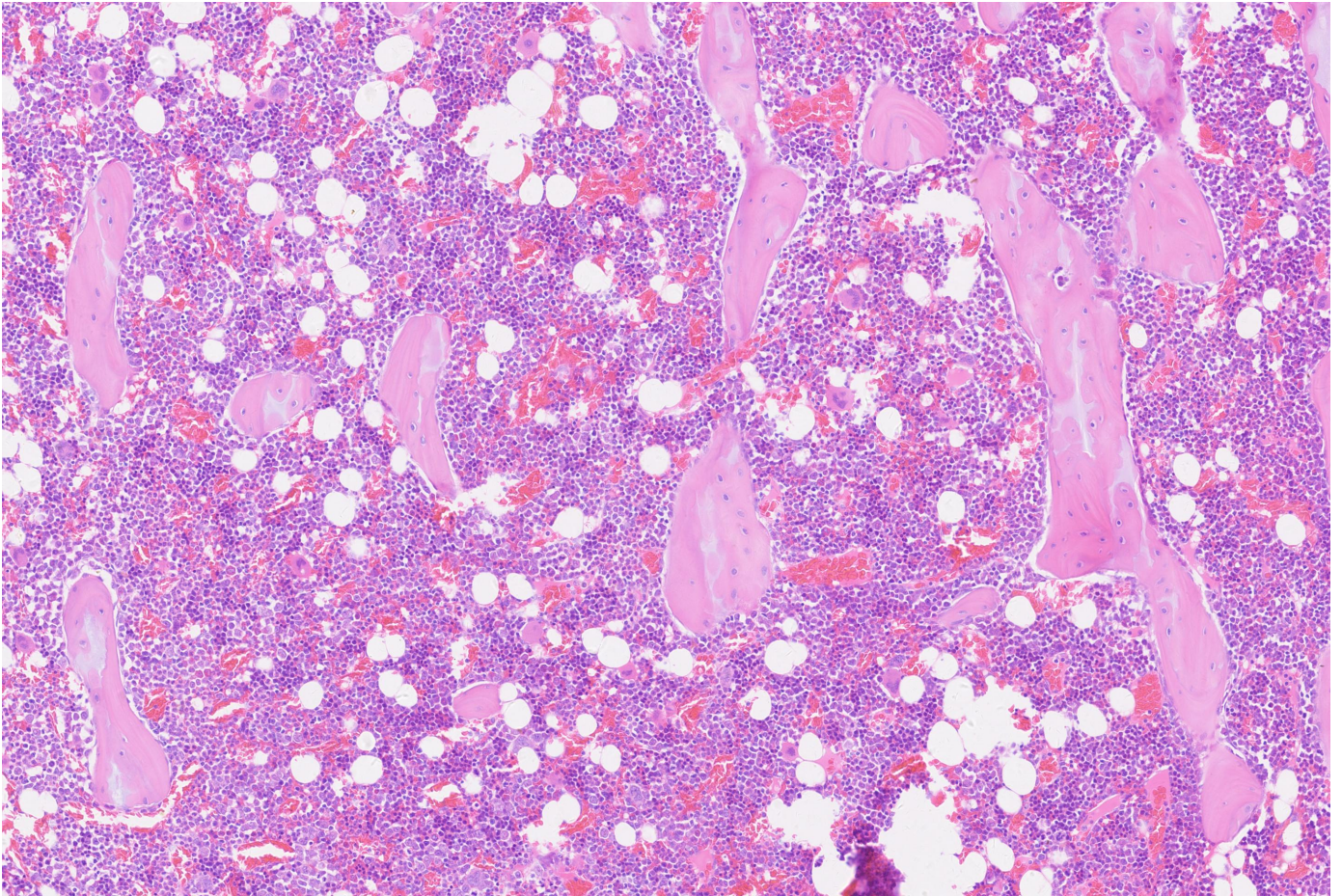

7.5mg/kg

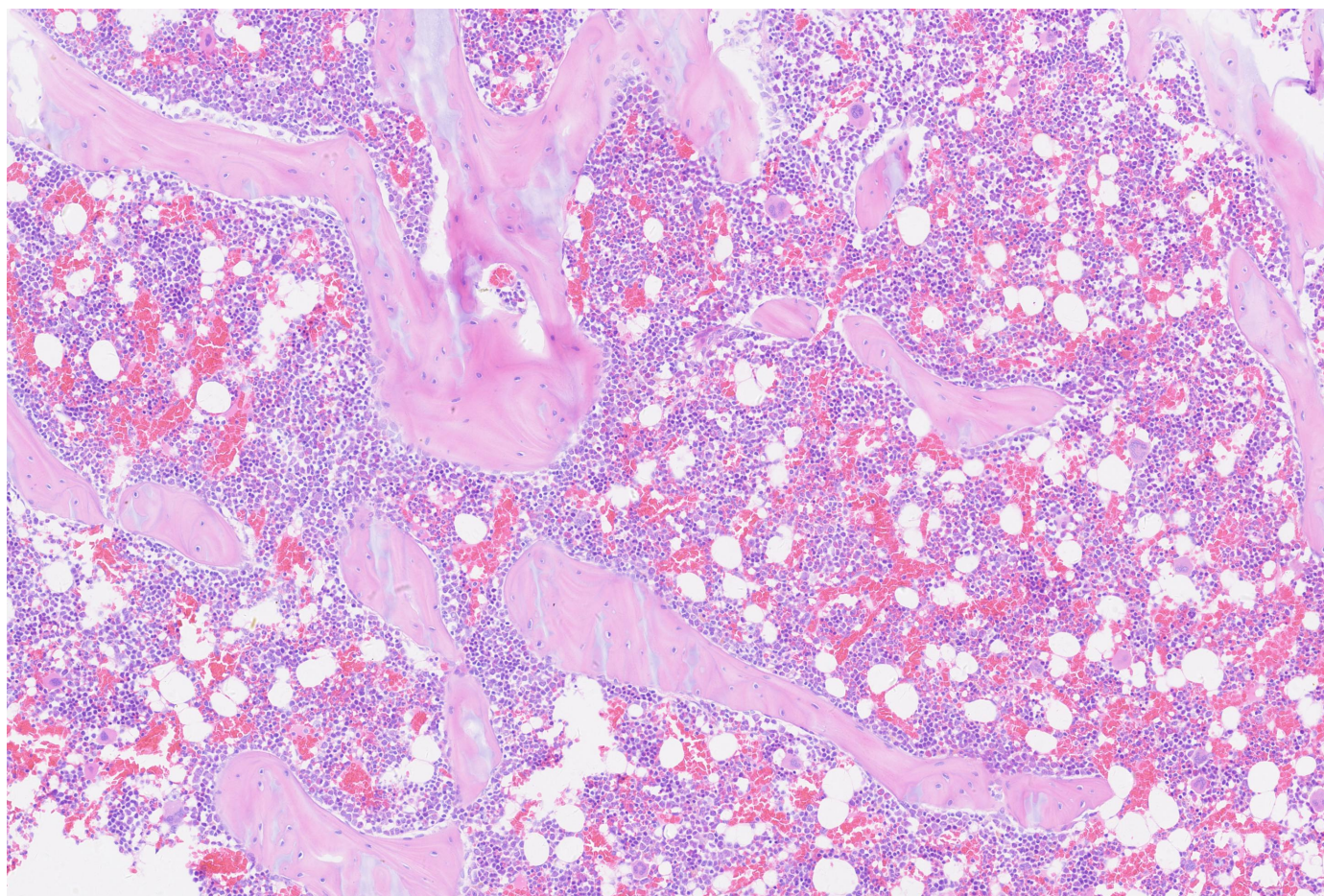

10mg/kg

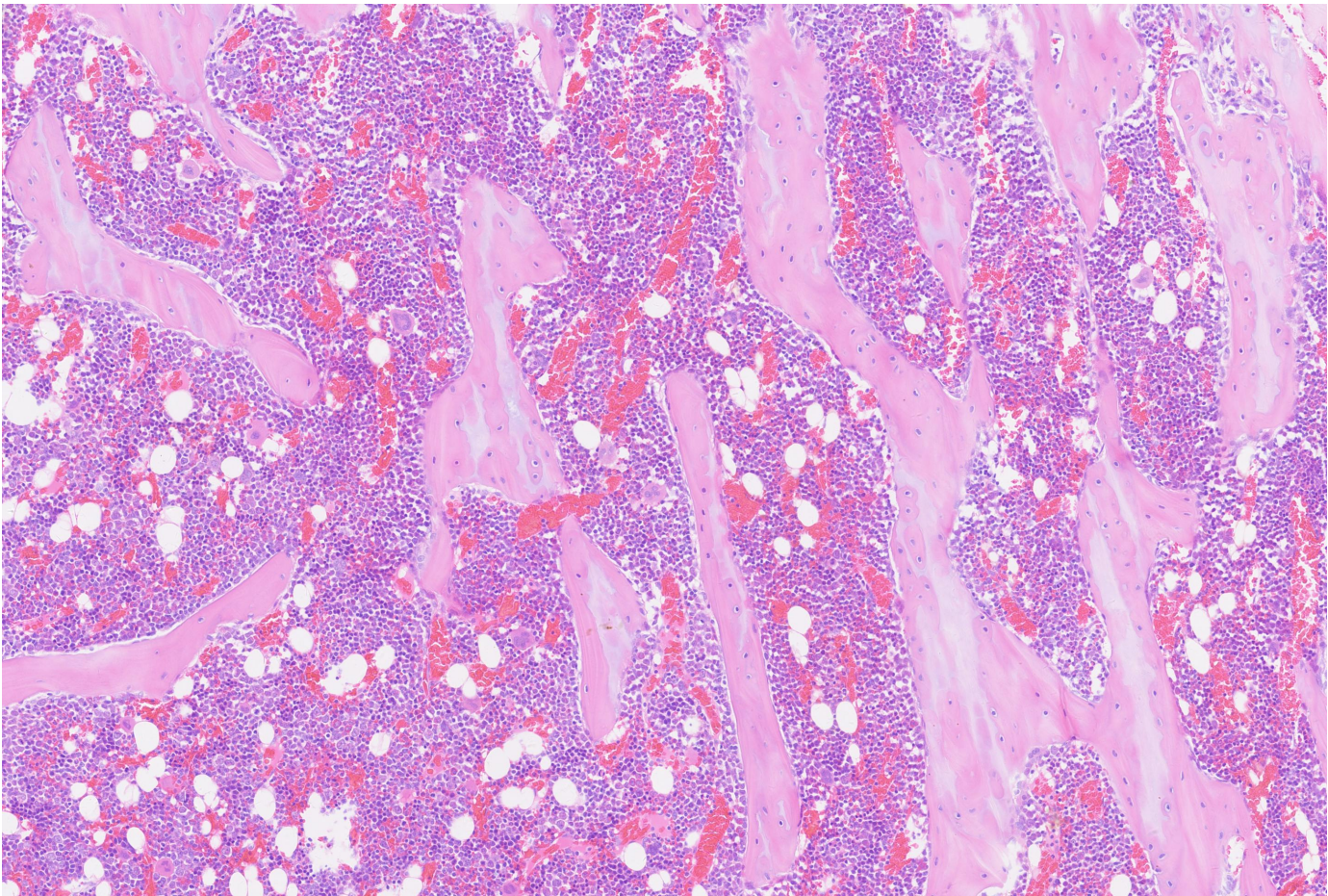

Supplement: Supplementary file 1 [file DataSheet1.pdf]

Shame

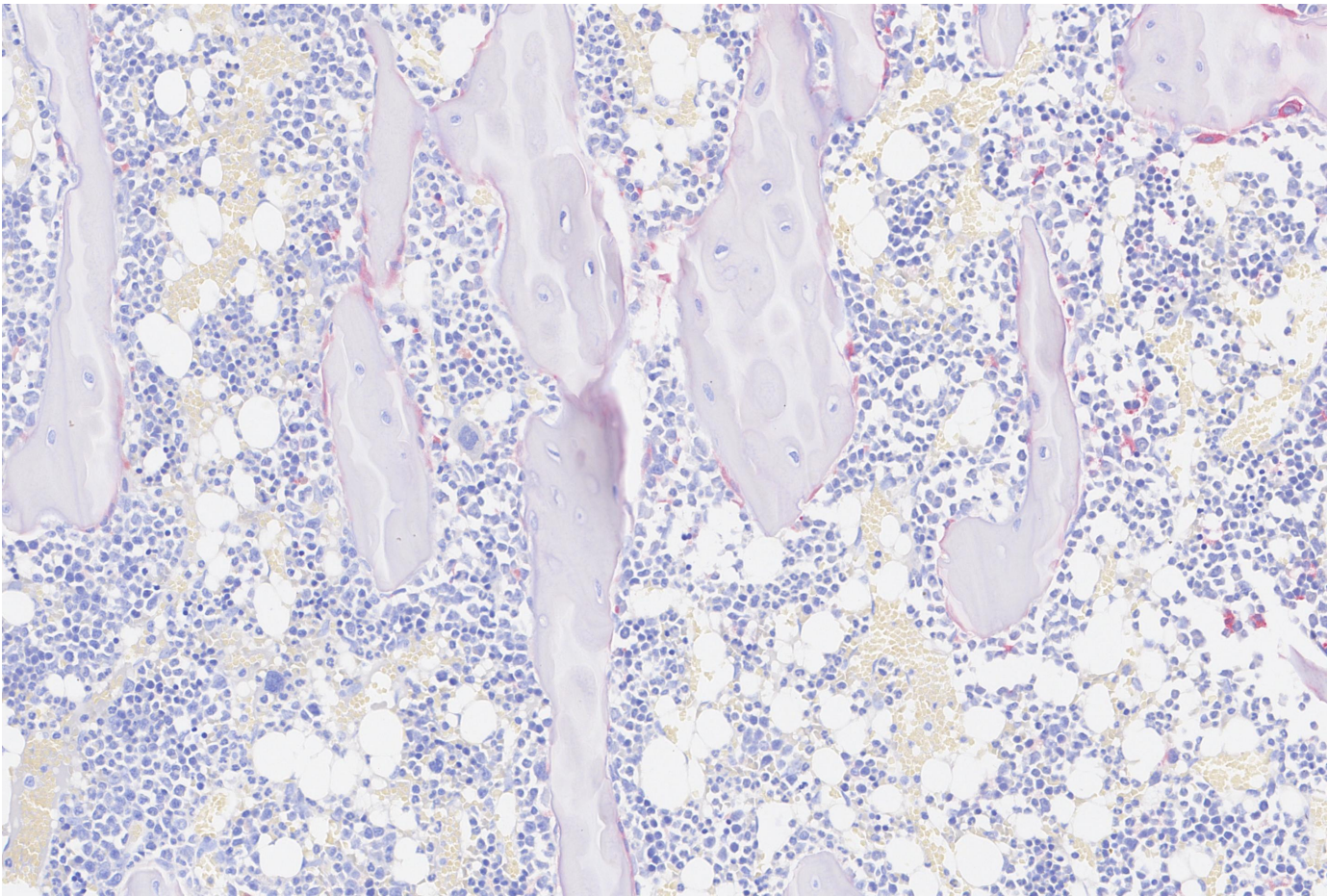

OVS

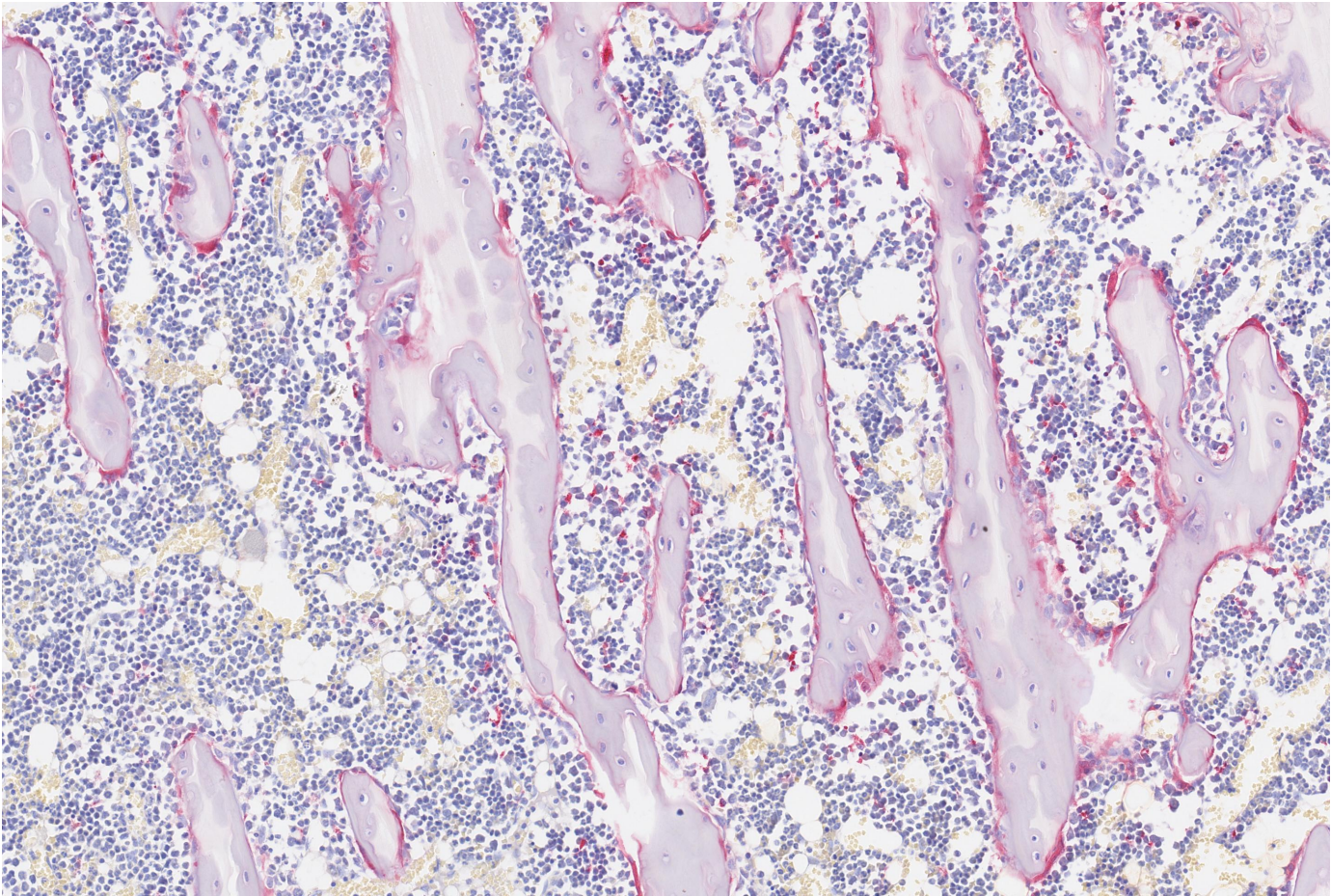

5mg/kg

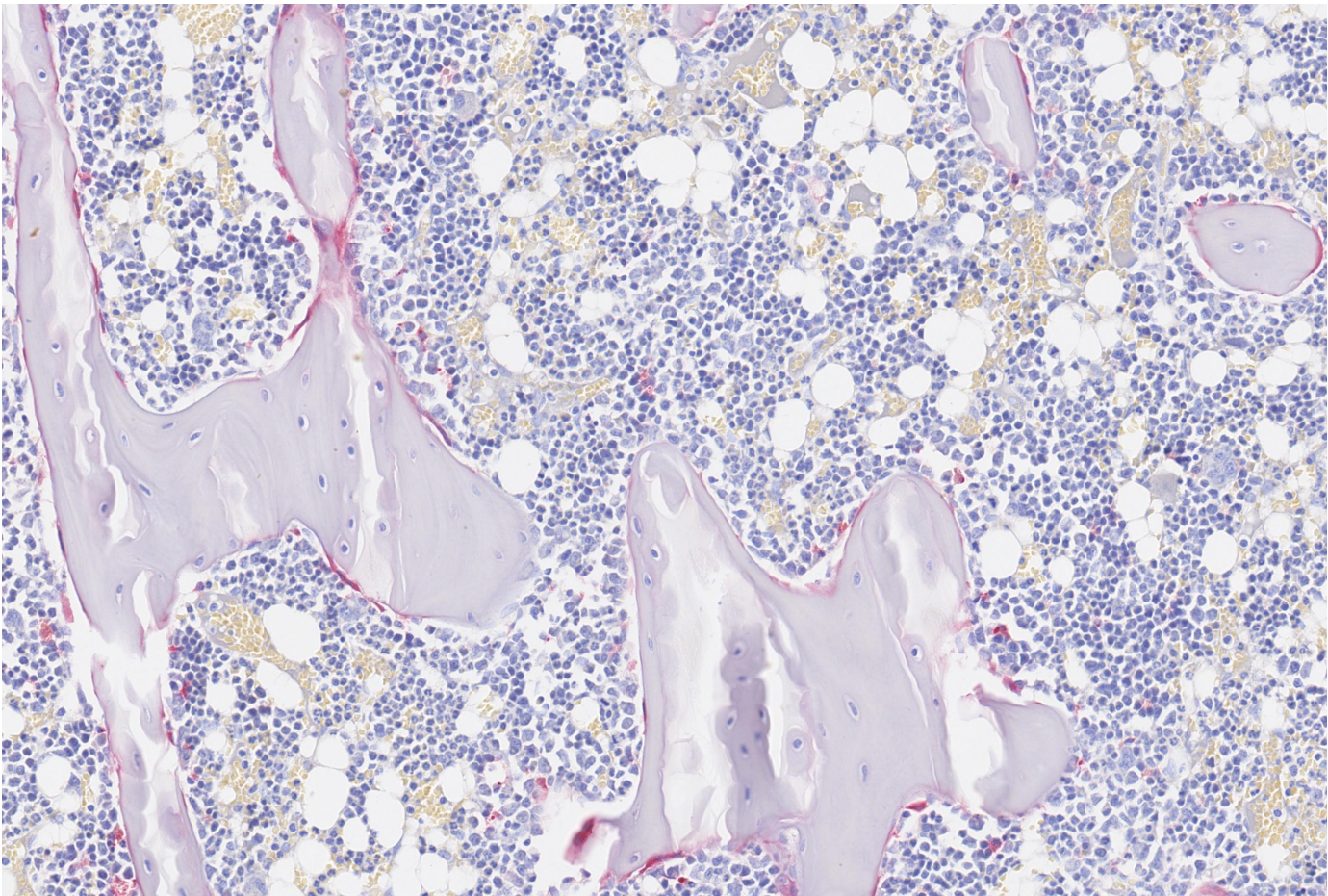

7.5mg/kg

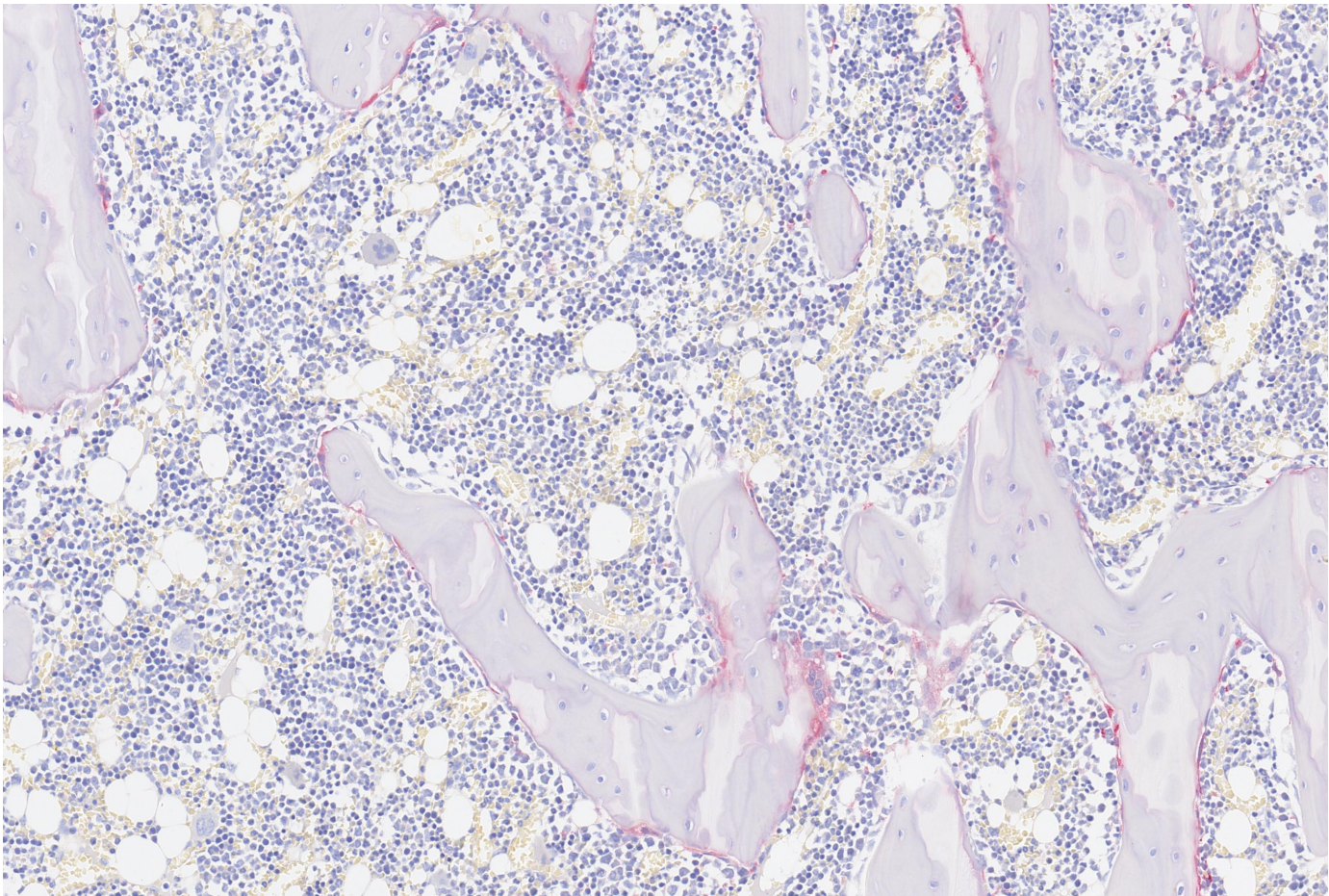

10mg/kg

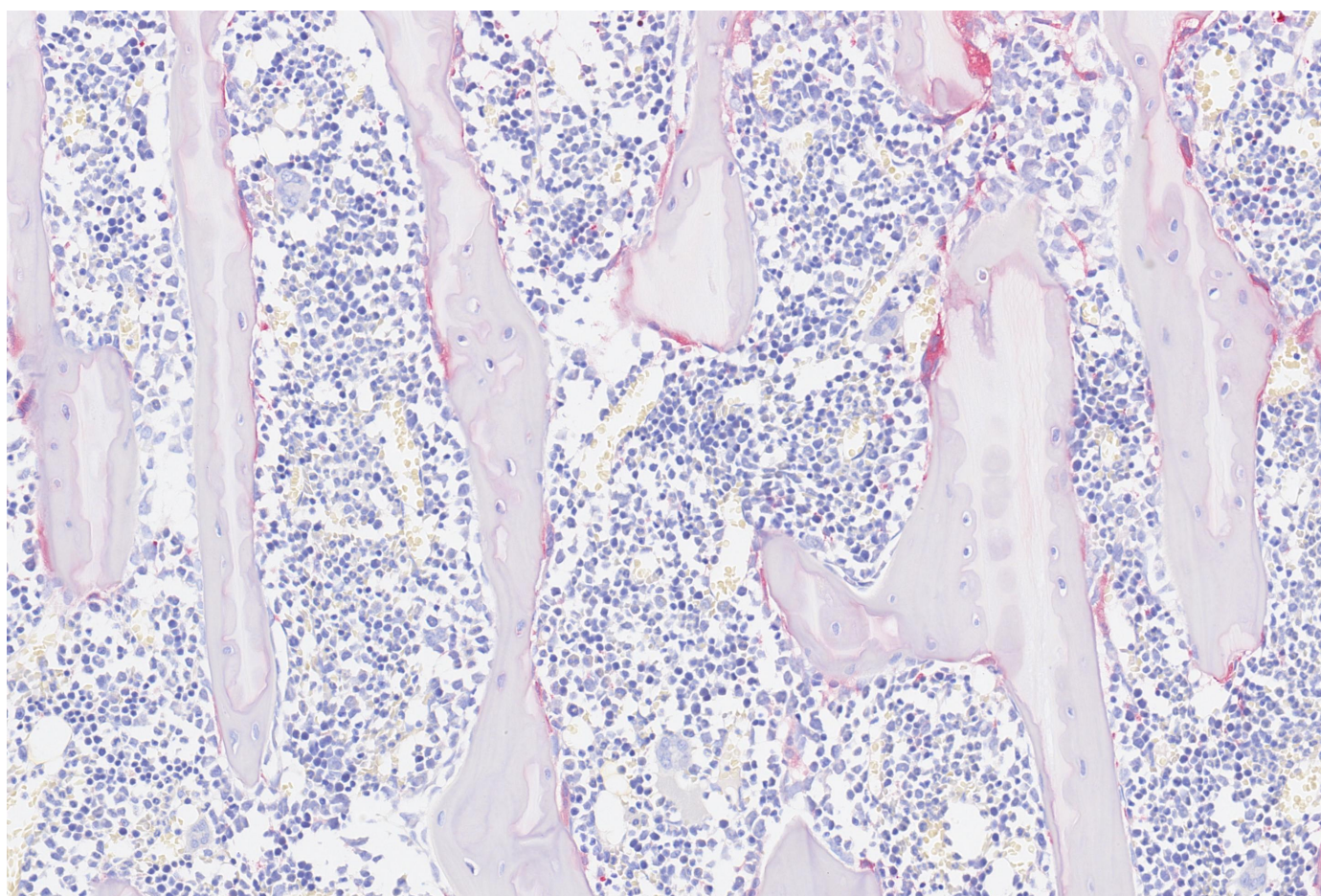

Supplement: Supplementary file 2 [file DataSheet2.pdf]
